# Behavioral dynamics of conversation, (mis)communication and coordination in noisy environments

**DOI:** 10.1038/s41598-023-47396-y

**Published:** 2023-11-20

**Authors:** Kelly Miles, Adam Weisser, Rachel W. Kallen, Manuel Varlet, Michael J. Richardson, Joerg M. Buchholz

**Affiliations:** 1 ECHO Laboratory, MU Hearing, and Performance and Expertise Research Centre, Macquarie University, Sydney, Australia; 2 ECHO Laboratory, Macquarie University, Sydney, Australia; 3 Performance and Expertise Research Centre, School of Psychological Sciences, Macquarie University, Sydney, Australia; 4 The MARCS Institute for Brain, Behaviour and Development, Western Sydney University, Sydney, Australia

**Keywords:** Human behaviour, Behavioural methods

## Abstract

During conversations people coordinate simultaneous channels of verbal and nonverbal information to hear and be heard. But the presence of background noise levels such as those found in cafes and restaurants can be a barrier to conversational success. Here, we used speech and motion-tracking to reveal the reciprocal processes people use to communicate in noisy environments. Conversations between twenty-two pairs of typical-hearing adults were elicited under different conditions of background noise, while standing or sitting around a table. With the onset of background noise, pairs rapidly adjusted their interpersonal distance and speech level, with the degree of initial change dependent on noise level and talker configuration. Following this *transient phase*, pairs settled into a *sustaining phase* in which reciprocal speech and movement-based coordination processes synergistically maintained effective communication, again with the magnitude of stability of these coordination processes covarying with noise level and talker configuration. Finally, as communication breakdowns increased at high noise levels, pairs exhibited *resetting behaviors* to help restore communication—decreasing interpersonal distance and/or increasing speech levels in response to communication breakdowns. Approximately 78 dB SPL defined a threshold where behavioral processes were no longer sufficient for maintaining effective conversation and communication breakdowns rapidly increased.

## Introduction

The ability to hold a conversation in noisy environments such as a restaurant or nightclub is critical for human communication and social connection. Indeed, humans possess a remarkable ability to communicate even in the most challenging acoustic environments, juggling both verbal and non-verbal channels of communication, all whilst negotiating constraints imposed by physical, physiological and perceptual limits, and social and cultural norms. In other words, human communication requires individuals to coordinate numerous behavioral processes across multiple modalities and time scales, reciprocally modulating behavior to maximize comprehension in relation to environmental constraints that degrade the transfer of information and intent.

The sophistication of behavioral coordination during communication is supported by a large body of literature demonstrating how the act of conversing with another individual is characterized by a cascade of coordinated interpersonal behaviors. These include the alignment and synchronization over the course of a conversational exchange of the vocabularies^1^, accents^2^, speaking rates and patterns^3,4^, pronunciation (e.g., clear speech^5,6^) body postures^7,8^ and gaze patterns^9^ of interlocutors. Consistent with signatures of behavioral coordination, the strength of neural coupling between speakers and listeners has been shown to covary during successful communication^10,11^. Moreover, such interpersonal coordination processes are known to generate elevated feelings of social connectedness^12^, affiliation^13,14^, likeability and rapport^15,16^, as well as facilitating prosocial behavior^17–23^ and cooperative interaction^24^ in human societies in general^25^.

In difficult acoustic environments, defined here as an adverse signal-to-noise ratio (SNR), previous research has also demonstrated how people mitigate the effects of environmental noise. This is achieved by boosting speech intelligibility^26^ through increasing how loud they talk relative to the noise (e.g., the Lombard effect^27^), by reducing talking speed^28,29^, or simply by moving closer to each other^30,31^ which indirectly increases the speech level at the listener’s ears. When individuals are committed to continuing a conversation in a noisy environment and are not able to overcome the SNR challenge, mutual understanding is jeopardized, and successful communication is compromised. In such circumstances, pairs may engage in a verbal or non-verbal request for an other-initiated repair (OIR)^32,33^, which can disrupt conversational and social engagement in pursuit of rectifying potential communication breakdown^34^.

Collectively, research implies that interpersonal and social behavioral coordination processes that support effective communication are synergistic. This entails that different behavioral degrees-of-freedom self-organize via reciprocal compensation to ensure task success, whereby individuals reciprocally modulate concurrently (both consciously and unconsciously) numerous behavioral processes across multiple modalities and time scales to maximize comprehension and information flow^11^. Across the individual^35–37^ and interpersonal coordination literature^38,39^, *reciprocal compensation* refers to the interdependence of perceptual-motor units, movements, actions, or behavioral processes that ensure a behavioral system (i.e., individual, pair, or team of individuals) can adaptively react to unexpected task or environmental changes, perturbations, or events. Importantly, nearly all of the previous research, which explored the verbal and nonverbal coordination dynamics that occur between conversational partners, has been conducted under stationary (i.e., movement restricted) and nominal (typically comfortable) conversational conditions. Thus, while contemporary theory assumes that conversing individuals dynamically and reciprocally structure their ongoing behavior to maintain effective communication in real-world listening scenarios, no previous research has directly explored the synergistic, multiscaled interpersonal coordination processes that supports communication in every-day, noisy listening environments.

**Figure 1:**
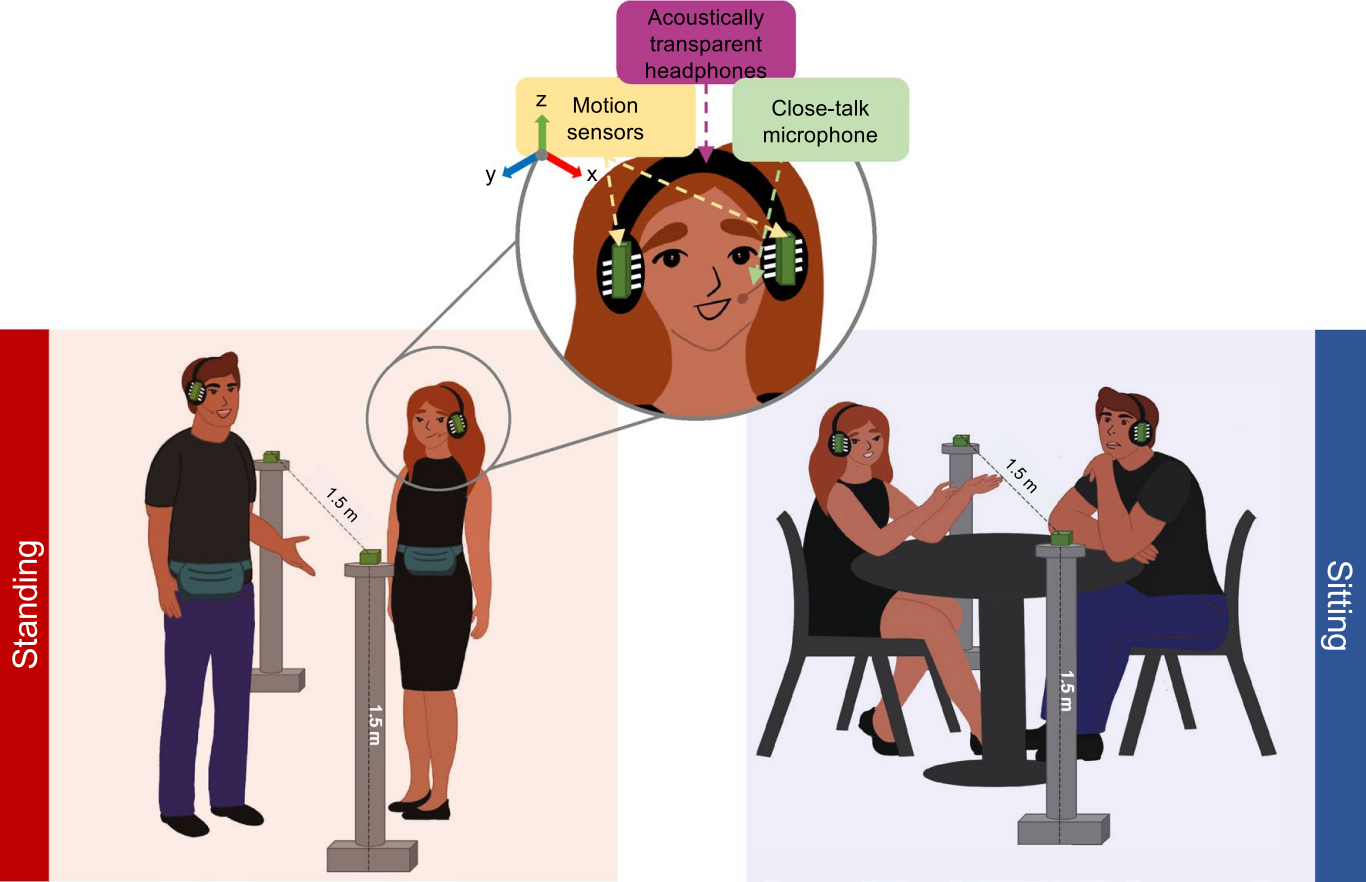
Illustration of the experimental setup. On the left, pairs were standing and free to move around while having an unrestricted conversation. On the right, pairs were seated during conversations. Acoustically transparent headphones delivered identical 3D simulated real-world acoustic environments to each person and motion sensors were attached to track head movement. Calibrated close-talk (boom) microphones recorded speech signals. Each person wore a pouch that housed the wireless receiver for the headphones and transmitter for the microphones.

Accordingly, the aim of the current study was to explore and identify synergistic coordination processes that occur between conversational partners when engaged in unrestricted conversations. This was achieved by recording the verbal and non-verbal behavior of conversing individuals in different physical and noisy environments. More specifically, realistic background noise replicating everyday communication scenarios, ranging from the relative quietness of a library (with a long-term average level of 53 dB SPL) to the bustling atmosphere of a party with music and conversations (with a long-term average level of 92 dB SPL) was presented binaurally (using non-individualized Head-Related Transfer Functions: HRTFs) to pairs of conversing individuals over ‘open’ headphones. Pairs were free to move about but were physically constrained to remain either in a seated or standing configuration (see Fig. 1) over the course of conversational trials. Each conversation lasted two minutes, as was the duration of the background noise. Participants were asked to return to their initial designated positions (far apart) and wait for a cue to initiate the conversation. Using motion-tracking sensors, we measured the dynamics of interpersonal distance and the reciprocal motor coordination that occurred between pairs. Additionally, we measured speech level dynamics using audio recordings, as well as identified communication breakdowns (indexed as other-initiated repairs; see Methods for more details).

Of particular interest was identifying how conversational partners maintain connection with each other when communication becomes difficult and inevitably breaks down. In pursuit of this, we observe the multimodal and synergistic processes pairs employ to *establish*, *maintain*, and *re-establish* communication in the noisy listening environments and following communication breakdowns. We theorize that processes to maintain successful communication in challenging background noise are derived through reciprocal compensation, such that pairs react to changes in the other given the demands of the environment^38^. As such, we anticipate that interpersonal synergies would manifest across modalities, where increasing background noise will drive pairs closer to each other and increase speech levels, and the stability of interpersonal motor coordination will strengthen. In addition, we expect that the physical barrier imposed by the table when pairs are seated will greatly impact interpersonal synergies. Given that the pairs cannot physically move closer to each other when seated across the table, we expect the magnitude and stability of interpersonal motor coordination to increase more in the seated condition compared to standing as the level of background noise increases.

Consistent with these expectations, three interrelated phases of behavioral modification and coordination were observed between conversational partners. First, *transient behavior* manifested at the onset of background noise and involved initial, rapid adjustments to enable a ‘baseline’ level of successful communication. Second, *sustaining behavior* perpetuated after the initial transient adjustments had abated comprised subtle, yet continuous, movement coordination processes and adjustments of speech level throughout conversation. Finally, conversation partners showed reactive or *intermittent resetting behaviors*, corresponding to a marked reduction of interpersonal distance and/or a marked increase in speech level following a communication breakdown. Accordingly, the below results are presented with respect to these three phases, in turn.

## Results

### Transient phase—initial behavior to set up for communication success

Following the onset of background noise, the occurrence of transient communication behaviors among conversational partners was observed—those reciprocal behavioral compensations aimed at establishing initial communication success. Here, we focus on the adjustments in interpersonal distance and speech level that followed the presence of background noise, as these adjustments contribute most to enhancing speech intelligibility. As predicted, individuals promptly reduced their interpersonal distance while simultaneously raising their speech level in response to increasing background noise levels.

**Figure 2:**
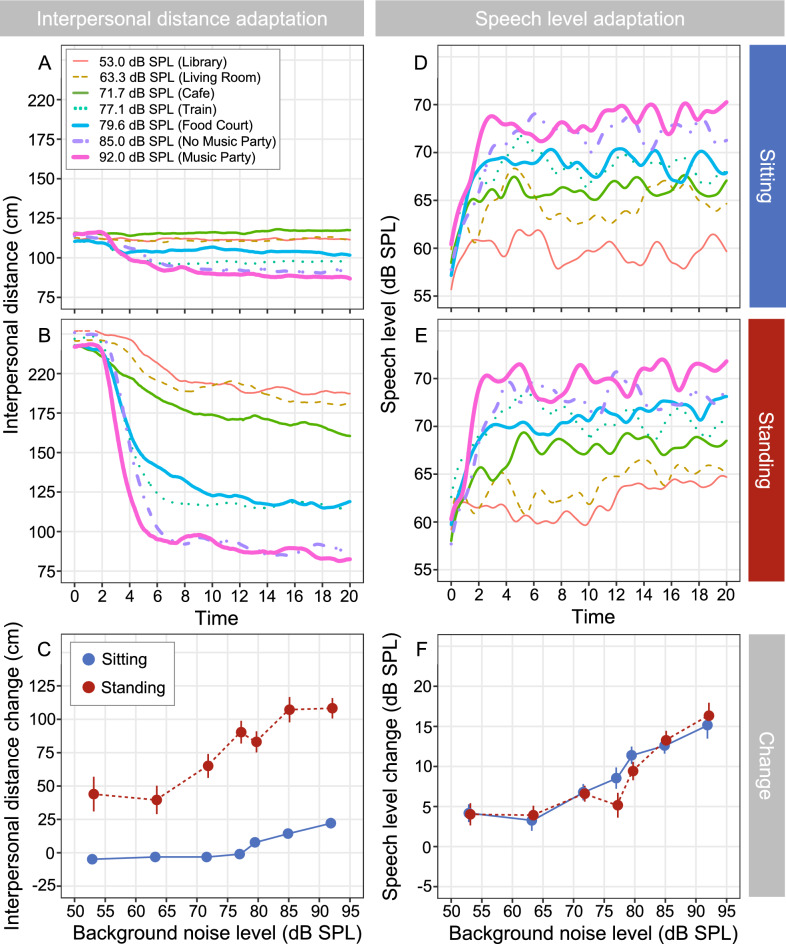
The adaptive behavior of interpersonal distance (**A** and **B**), and speech level (**D** and **E**) as a function of time with background noise and talker configuration as parameters. (**C**) and (**F**) illustrate the mean change (error bars depict standard errors) in interpersonal distance and speech levels (the difference between the final and starting states).

*Interpersonal distance adjustments:* The variation in interpersonal distance of pairs of communication partners was assessed over the first 20 s following the onset of each background noise level (Fig. 2A). Communication partners rapidly adjusted their interpersonal distance by moving closer to each other within the first 5 to 10 s of their conversation (Fig. 2A and B). This adjustment depended on the level of background noise and was less pronounced when partners were seated, compared to when they were standing, due to the physical constraint imposed by the table. The change in interpersonal distance was calculated as the difference between the final and starting states of the interpersonal distance, where 0 cm corresponds to no-change, and positive values indicate a reduction in interpersonal distance. Visual inspection of the data (Fig. 2C) suggests a larger effect of background noise on interpersonal distance for standing compared to seated pairs of conversational partners, with a significant interaction between background noise and talker configuration in terms of the change in interpersonal distance for seated compared to standing pairs (F (1, 283) 21.08, *p* < 0.001, η_p_^2^ = 0.07). Specifically, a linear increase in change in interpersonal distance was evident as the level of background noise increased, with a change in interpersonal distance of 0.70 cm (standard error, SE: 0.20 cm; confidence interval, CI: 0.30 cm, 1.09 cm) when seated, and 2.0 cm (SE: 0.20 cm; CI: 1.61 cm, 2.40 cm) when standing, for every 1 dB increase in the level of background noise. The smaller change in interpersonal distance with increasing level of background noise in the seated configuration likely arose because pairs were initially positioned closer together compared to the standing condition and could only reduce interpersonal distance by leaning forward over the table.

*Speech level adjustments:* As with interpersonal distance, conversational partners also rapidly adjusted their speech levels in response to increases in background noise (Fig. 2D and E) with a significant main effect of background noise on change in speech levels (the difference between final and starting states) (F(1, 277) 123.18, *p* < 0.001, η_p_^2^ = 0.31). Note that the onset of the speech levels (the zero-time point) was not taken at the time of onset of the background noise but rather from the onset of the speech utterance (see Methods for more details). Interestingly, there was no credible evidence of talker configuration on increases in speech levels (F (1, 277) 0.418, *p* = 0.59, η_p_^2^ = 0.001). Rather, speech levels increased by a mean of 0.32 dB (SE: 0.03; CI: 0.26, 0.38) for every 1 dB increase in background noise level whether talkers were seated or standing. That is, regardless of whether pairs were seated or standing while talking to each other, conversational partners set themselves up for communication success by rapidly adapting speech levels in response to the increasing noise demands.

**Figure 3:**
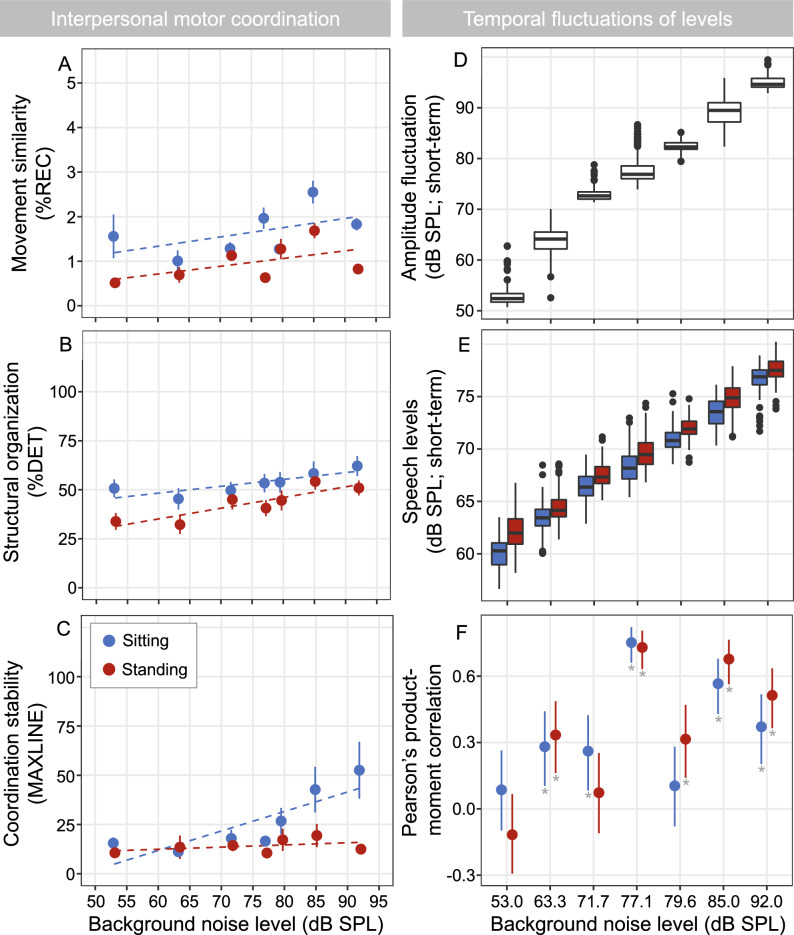
(**A**, **B** and **C**) show the mean and standard error of the three main interpersonal motor coordination measures of conversational partners as a function of background noise level and talker configuration, with dashed regression lines to show statistical modeling. (**A**) shows the similarity of movement (%REC), (**B**) the structural organization (%DET), and (**C**) the stability of coordination (MAXLINE). The right panels illustrate the temporal fluctuations across background noise levels and talker configurations where appropriate. (**D**) is a boxplot of the amplitude fluctuation of the background noise, (**E**) shows a boxplot of the amplitude fluctuation of the median speech levels and (**F**) depicts Pearson’s product-moment correlations between the background noise envelope (i.e., level fluctuations) and the time-aligned median speech envelope with error bars denoting 95% confidence intervals and those marked with asterisks highlighting significant p-values.

It should be noted that decreasing the interpersonal distance results in an increase in the effective (receiver-related) speech level at the listeners’ location, an effect that is in addition to the source-related speech level adjustments shown in Fig. 2 (right column). Assuming a 6 dB increase in level with each halving in distance, this results in an overall distance-related benefit (i.e., speech level increase) of up to 2.2 dB in the sitting configuration and up to 9.0 dB in the standing configuration (as observed in the loudest, music party environment). We previously showed a significant effect of talker configuration on the receiver-related speech level—an effect barely seen in the (source-related) speech levels shown in Figs. 2 and 3 here^30^. When standing, the receiver-related speech levels were up to 3.0 dB lower in the quietest environments and up to 3.4 dB higher in the loudest environments. Moreover, these results highlighted that despite the different compensatory mechanisms, the effective (receiver-related) SNR at the listener’s location still decreased substantially over the 39 dB range of considered noise levels, by about 21.2 dB when sitting and about 16.0 dB when standing.

### Sustaining phase—reciprocal coordination processes to support ongoing conversations

After the initial, transient adjustments in interpersonal distance and speech level, both remained relatively stable over the remaining course of conversational trials (Fig. 2A,B,D,E). This, however, did not mean that the speech levels and movements of the conversing individuals was static. On the contrary, both speech levels and movements continued to fluctuate over the course of the conversational trials, with these subtle fluctuations reflecting the behavioral coordination process that pairs employ to effectively sustain and facilitate ongoing communication. Importantly, we observed that interpersonal movement and speech level variation were influenced by increases in environmental noise. Moreover, the effects of environmental background noise on the occurrence and stability of interpersonal coordination was modulated by the differential physical movement constraints that characterized our seated and standing configurations.

*Interpersonal movement coordination:* To determine whether and how interpersonal motor coordination processes covaried with the level of background noise and whether talkers were seated or standing, we quantified the magnitude and stability of the postural (body) movement coordination (as measured by participants’ center-of-head position over time) that emerged between pairs of conversational partners using Cross-Recurrence Quantification Analysis (CRQA). CRQA determines the dynamic similarity (recurrence) or covariance of two time-series trajectories independent of dynamics of underlying data. Because CRQA is highly sensitive to the subtle space–time correlations that can occur between two motion trajectories (see^40–42^), it is ideal for assessing complex patterns of interpersonal movement or postural coordination^7^, including during conversation (e.g.,^7,43,44^; see Methods for more details). CRQA generates a range of recurrence quantification or covariance metrics. Of interest to us were the primary CRQA metrics: percent recurrence (%REC), percent determinism (%DET), and MAXLINE. %REC reflects the percentage of states that are recurrent (close together) across the two movement trajectories and therefore provides a measure of overall movement similarity. %DET captures the amount of recurrent activity that forms sequences of recurrent states and therefore reflects the degree to which the sequential patterning of similar movements is deterministic, as opposed to being random. MAXLINE measures the maximum number of consecutive states that remain close together (are recurrent) over time (i.e., the longest sequence of recurrent states between two movement trajectories in reconstructed phase space), reflecting stability of coordination^7,43,45,46^. Higher values reflect higher magnitudes of movement coordination (%REC), more structured patterns of movement coordination (%DET), and more stable interpersonal coordination (MAXLINE). For ease of interpretation, we refer to %REC as *movement similarity*, %DET as *structural organization*, and MAXLINE as *coordination stability*.

CRQA analysis revealed that background noise significantly affected both movement similarity (F (1, 283) 6.55, *p* = 0.01, η_p_^2^ = 0.02;) and structural organization (F (1, 283) 28.821, *p* = 0.01, η_p_^2^ = 0.08) of coordination (Fig. 3A and B), indicating that movements of pairs of conversational partners became more coordinated as the level of background noise increased. Specifically, for every 1 dB increase in background noise level, conversational partners increased the similarity of their movements by 0.016% (SE: 0.006%; CI: 0.004%, 0.03%) and the structural organization of their movement coordination by 0.45% (CI: 0.25%, 0.62%).

The configuration in which conversations took place—seated or standing—also influenced how much conversational partners coordinated their movements in terms of movement similarity (F (1, 283) 28.024, *p* < 0.001, η_p_^2^ = 0.09) and structural organization (F (1, 283) 22.944, *p* < 0.001, η_p_^2^ = 0.07) of coordination. Interestingly, movement similarity was, on average, greater when conversing pairs were seated around the table (1.65%; SE: 0.15%; CI: 1.33%, 1.96%) compared to when they were standing (0.84%; SE: 0.15%; CI: 0.53%, 1.16%). Similarly, the structural organization of coordination was also greater when pairs were seated (53.3%; SE: 3.0%; CI: 47.1%, 59.5%) compared to standing (43.0%; SE: 3.0% CI: 36.8%, 49.2%). Together, these results indicate that the movement of pairs is more coordinated when seated compared to when standing, likely because standing pairs could move closer together and thus there was less need (and space) for them to coordinate their ongoing movements to sustain effective conversation compared to when seated. Consistent with this, we found main effects of background noise (F (1, 283) 18.66, *p* = 0.011, η_p_^2^ = 0.06) and talker configuration (F (1, 283) 15.32, *p* < 0.001, η_p_^2^ = 0.05) on the stability of coordination (i.e. MAXLINE), as well as a significant interaction between background noise and talker configuration (F (1, 283) 11.93, *p* < 0.001, η_p_^2^ = 0.04). Pairs of conversational partners maintained relatively unvarying coordination stability over the entire range of background noise levels while standing but exhibited high stability of coordination in the loudest background noises when seated (Fig. 3C). Specifically, coordination stability increased by 0.99% [CI: 0.64%, 1.34%] for every 1 dB increase of background noise level when pairs were conversing, but only while seated. Again, this indicates that movement coordination is less important in mitigating the effects of noise on communication when conversational partners are standing, given the greater possibility for initial (transient) and intermittent changes in interpersonal distance (i.e., resetting; see below) when standing compared to when seated.

**Figure 4:**
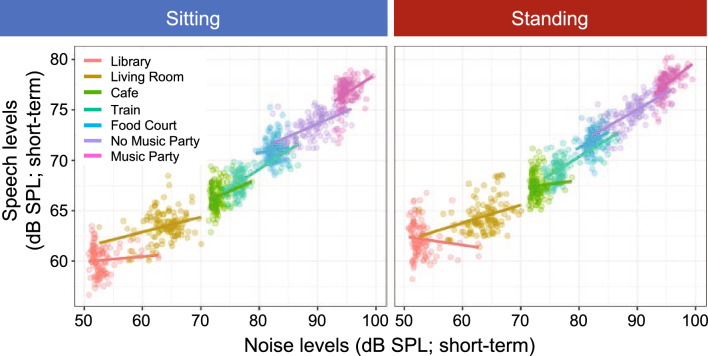
Scatterplots showing the relationship between the short-term background noise level and speech levels.

*Speech level variations:* To examine how individuals adjust their speech levels in response to the level fluctuations of the background noise, we calculated short-term broadband sound levels within 2 s-long time windows to derive the temporal envelopes of each noise signal as well as of the corresponding median speech level across all subjects, calculated using the same time windows (Fig. 3D and E). Some environments (corresponding to those with longer whiskers on boxplots) showed greater variability in amplitude fluctuations—the living room (63.3 dB SPL), the train station (77.1 dB SPL), and no music party (85 dB SPL). Pearson’s product-moment correlations between the time-aligned envelopes of the background noises and speech signals are plotted as a function of background noise and talker configuration (Fig. 3F). The highest correlation in modulation of speech levels within pairs of conversational partners occurred when a delay of approximately 2 s was removed from the speech signal, suggesting that talkers required about 2 s to adjust their speech level to changes in noise level (i.e., the background noise to speech level synchrony operated at an approximately 2 s lag—see Supplementary Information Table 1 for Pearson’s product-moment correlations and their corresponding slopes, and Fig. 4 for graphical representation of the relationship between the short-term background noise level and speech levels). That is, SNR challenges prompt conversational partners to adapt their speech levels in response to changing background noise levels with about a 2 s delay.

### Resetting phase—intermittent behavior to help restore communication when things go wrong

Communication breakdowns in typical conversation occur around once every 5 min—at least in terms of an other-initiated repair being signaled by a listener^32^. Here we examined how background noise and seated versus standing conversations impacted communication breakdowns during conversations. We also quantified the reciprocal resetting behaviors pairs used to resolve these breakdowns. We use the term ‘resetting’ as the objective is to re-establish a new baseline level of behavior to better support future conversational interaction and minimize future communication breakdowns.

**Figure 5:**
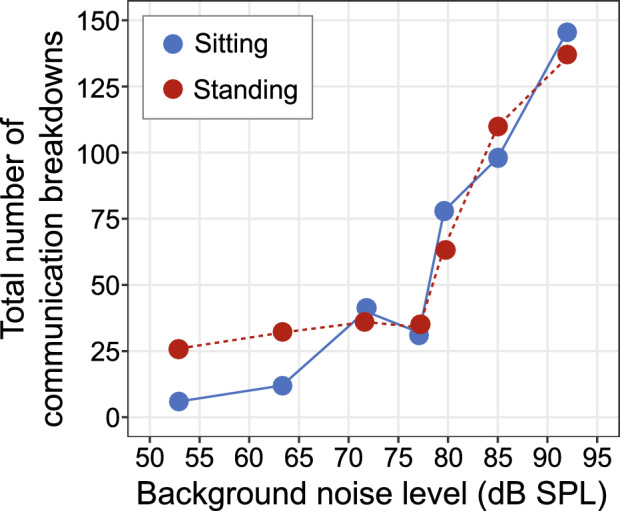
Total number of communication breakdowns while participants were in the seated or standing configuration across different levels of background noise.

The total number of communication breakdowns summed across all participants (Fig. 5) per noise environment over the entire duration of the conversation revealed an increase in the number of breakdowns as background noise level increased. Below 78 dB SPL (i.e., within the four quietest environments) the number of breakdowns remained relatively low and largely consistent across noise levels. Above 78 dB SPL, however, communication breakdowns steeply increased with noise level. Accordingly, we separately analyzed the number of communication breakdowns from 78 dB SPL and above, as this level appears to be a critical noise level where compensatory coordination and speech level mechanisms began to fail.

Analyzing only the three background noise levels above 78 dB SPL, we found a significant main effect of background noise (F (1, 151) = 40.65, *p* < 0.001, η_p_^2^ = 0.21), no credible evidence of a main effect of configuration, i.e. seated or standing (F (1, 151) = 0.042, *p* = 0.838, η_p_^2^ < 0.001), and no interaction (F (1, 151) = 0.008, *p* = 0.926, η_p_^2^ < 0.001). Specifically, for every 1 dB increase in background noise level, the number of communication breakdowns increased, on average, by 0.15 (SE: 0.024; CI: 0.107, 0.203). This corresponds to an increase of one communication breakdown for every 7 dB increase in noise level, with an average of 1.2 communication breakdowns per minute.

As a matter of completeness, we also ran the same analyses looking at the quieter environments. Similar to the louder environments, we found a significant main effect of background noise (F (1, 107) = 9.18, *p* = 0.003, η_p_^2^ = 0.079), but there was an additional main effect of talker configuration (F (1, 107) = 4.243, *p* = 0.042, η_p_^2^ < 0.038) and no interaction term (F (1, 107) = 2.36, *p* = 0.11, η_p_^2^ < 0.017). For every 1 dB increase in background noise level, the number of communication breakdowns increased, on average, by 0.02( SE: 0.008; CI: 0.009, 0.04), with an average of 0.3 communication breakdowns per minute.

Analysis of the pairs’ behavior at the locus of communication breakdowns revealed significant changes in interpersonal distance and speech level in response to breakdown. Pairs reciprocally compensated by reducing their interpersonal distance (F (1, 791) = 8.095, *p* = 0.005, η_p_^2^ = 0.01) and significantly increased their speech levels (F (1, 787) = 71.29, *p* < 0.001, η_p_^2^ 0.08) following a communication breakdown. Averaged across the levels of talking configuration and background noise, pairs moved 5 cm closer, and the talkers’ speech levels increased by 3.2 dB in response to the signaling a communication breakdown. Both resetting behaviors captured here—move closer, talk louder—act to increase the SNR at the listener’s ear.

## Discussion

The ability to follow a conversation in noisy environments is critical to successful communication and social interactions. The current study explored the synergistic coordination (reciprocal compensation) processes employed by conversational partners to establish, maintain, and re-establish effective communication and minimize communication breakdowns when conversing in noisy environments. More specifically, we assessed the movements, speech levels and communication breakdowns of pairs of talkers conversing in different levels of background noise, with this analysis revealing three dynamic phases of adaptive behavior, which we define as *transient*, *sustaining*, and *resetting* behavioral processes.

We identified multimodal processes of reciprocal compensation that the pairs used to hear and be heard when establishing and maintaining communication, and when re-establishing communication after breakdown. These reciprocal processes entailed modifications in interpersonal distance, postural movement coordination and speech level that synergistically compensated for changes in background noise level and environmental constraint (i.e., standing versus seated at a table). The data demonstrate that even though conversational partners were free to make adjustments to hear and to be heard, these adjustments only go so far to compensate for the background noise. We identified relatively few communication breakdowns when background noise was lower than 78 dB SPL, suggesting that behavioral adaptations and coordination served to facilitate speech intelligibility below this level of background noise. Above 78 dB SPL, however, communication breakdowns significantly increased (Fig. 5) suggesting that there may be a critical noise level above which behavioral processes are unable to fully compensate for the poor SNR conditions. Crucially, these environments are common in everyday social interactions (e.g., meetings with clients in busy cafes; dinner with the family in a busy restaurant).

Transient behavioral processes occurred immediately following the onset of background noise and manifested as rapid and proportional adjustments in both interpersonal distance and speech levels (Fig. 2). Unsurprisingly, the greatest transient change in initial interpersonal distance occurred when pairs of conversational partners were free to move around while standing and background noise level was loudest. Given the physical barrier of the table restricting motion, interpersonal distance adjustments were minimal when pairs were seated and conversing in the quietest background noise. Although initial speech levels also increased as background noise increased, speech levels were surprisingly robust to the imposed physical constraint of the table, with similar speech level adjustments observed when pairs were both seated and standing. Combined with the finding that pairs’ sustaining behavior entailed more movement coordination when seated compared to when standing, this might indicate that individuals preferentially employ movement-based compensatory behaviors over changes in speech level as background noise levels increase. Indeed, while we observed increasing speech levels were observed as the level of background noise increased—no doubt important for effective communication—minimizing changes in speech level by simply moving closer together or coordinating one’s movements with a conversational partner might be preferable to raising one’s voice in many social settings.

With respect to the subtle, behavioral processes that operated to sustain communication following transient adaptations, we observed that both movement similarity and the structural organization of interpersonal motor coordination increased as background noise level increased. This is consistent with evidence that interpersonal coordination is greater when pairs of conversational partners converse in loud simulated traffic noise (90 dBA) compared to quiet^47^. Similarly, pairs coordinated their movements when conversing in speech-shaped noise at 78 dB SPL, but significantly less so at 54 dB SPL^48^. Note that 78 dB SPL happens to correspond to the estimated critical threshold of communication breakdowns we report here. It is also consistent with other reports that are suggestive of conversational difficulty, indexed by overlapping turn-taking, which is significantly greater at 78 dB SPL compared to 72 dB SPL^49^. Together, the data strongly suggest that at least for young, typical hearing adults, 78 dB SPL might reflect a critical, real-world threshold for difficult conversations.

The movement similarity and structural organization of interpersonal motor coordination were significantly greater when pairs were seated compared to standing (e.g., Fig. 3A and B), and this was likely due to the physical constraint of the table in the seated configuration limiting the magnitude of interpersonal distance adjustments. This, in turn, required individuals to coordinate their head and body movements more closely to reciprocally compensate for increases in background noise, consistent with evidence that imposing the same physical constraint on pairs during conversation leads to increasingly synchronized coordination of behavioral responses^50^. Surprisingly, movement coordination only became more stable (greater MAXLINE) as background noise increased in the seated configuration (Fig. 3C); i.e. only when the pairs were restricted in their physical movement. While this indicates that the stability of movement coordination is modulated by interpersonal distance, it also implies that sustained coordination might be operating as interpersonal synergy to signal that one is committed to the challenge of conversing in background noise (i.e., a pro-social signal to continue conversing through adversity). Moreover, it functions to facilitate communication by assisting with tracking the signal in the noise^51^. A subsequent possibility that could be explored in future work is that interpersonal motor coordination may serve as a metric of listening difficulty^48^ and/or communication effort.

Pairs also attempted to sustain communication by varying their speech level in response to the background noise level and variation (e.g., Fig. 3, right panels). A significant component of the variation is derived from the inherently different fluctuations of the various environments^52^. Some environments have greater fluctuations (e.g., living room, train station, no music party) while others are rather stationary. Manifested in the correlation between the fluctuations of the background noise and speech levels, the strength of this co-modulation peaked when the pairs were conversing in the highly fluctuating train station environment (e.g., Fig. 3F). Crucially, a distinct pattern is evident which captures this relationship; correlations are high in the highly fluctuating train station environment, low in the louder yet stationary food court environment, high again in the louder highly-fluctuating no-music party environment, and lower in the loudest, but rather stationary music party environment. In addition to the extent of the noise level variations, the overall noise level drives the need for pairs to adjust their speech level. As such, the living room is highly modulated but rather quiet, which resulted in only minor though still significant speech level adjustments (as indicated by the rather shallow slopes in Fig. 4). These results provide further evidence that talkers react to fluctuations in broadband noise level by adapting their instantaneous speech level. This is in line with studies that demonstrated local (i.e., short-term) adaptation effects to temporal modulation variation in background noise, but which were generally tested under less realistic conversational conditions^53–55^.

Finally, when communication breakdowns inevitably occurred, pairs exhibited resetting behaviors. We showed that following the signaling of a communication breakdown, pairs moved on average 5 cm closer to each other, quantifying the often reporting ‘leaning forward’ effect following the signaling of a breakdown^56,57^. It is also possible that this movement was coupled with additional compensatory mechanisms such as head turning and ear cupping^58^ which may be serving as a gain mechanism to boost the speech signal at the ear^59^. We also observed an increase of 3.2 dB in the talker’s average speech level in response to the listener signaling a communication breakdown, which is a common, although rarely quantified, behavioral adjustment^60,61^.

Our reporting of communication breakdowns is a robust and direct metric for assessing real-world conversational difficulty during interactive communication. Previous studies have reported that incidental noise captured during otherwise quiet recording sessions (e.g., newspaper rustling, cooking) were more likely to result in a communication breakdown^32,62,63^. Here we systemically showed that increasing background noise levels increases communication breakdowns. While 78 dB SPL appears to be a critical noise level where compensatory mechanisms begin to fail, it should be highlighted that communicating in background noise is not purely an SNR challenge that can be resolved through behavioral and coordination adjustments. Physical, physiological, and psychological constraints must also be considered. As observed here with reference to when pairs were seated around a table, physical barriers can limit the ability to decrease interpersonal distance, but also speech levels have inherent physiological limitations, and psychological conventions (e.g., cultural and / or power dynamics) may further limit the proximity in which people feel comfortable conversing^64–66^ and / or how loudly they talk in certain environments^67^.

The current study provides the first comprehensive investigation of the behavioral processes individuals spontaneously employ to facilitate and sustain effective communication in the presence of realistic, everyday background noise. The results obtained are consistent with the theoretical assumption that the interpersonal and social behavioral coordination processes that support effective communication are synergistic, and provide clear evidence that individuals expertly coordinate and reciprocally adapt numerous behavioral processes across multiple modalities to maximize comprehension as a function of environmental constraint. Accordingly, the findings of the current study emphasize the importance of investigating interpersonal conversation and interaction—as well as human social interaction in general—as a complex, embedded dynamical system of synergistic, multiscaled behavioral processes^11,38,68^. However, it should be noted that the ability to generalize the results of the present study is limited by the degree to which its method can elicit conditions that resemble realistic settings. The differences between the two lies in the unnatural acoustical and technical setups, the way in which conversation was elicited and was being observed by the experimenters, and other factors that may be perceived by the participants^30^.

## Materials and methods

The experimental data and test methods are extensively reported in our previous paper^30^ and presented here in brief.

### Ethics statement

All participants provided written and informed consent before participating in this study. All experimental protocol used in this study—including the sharing of de-identified research data—were approved by the Macquarie University Human Research Ethics Committee (5201929708215, Project ID 2970) and were carried out in accordance with the enforced guidelines.

### Participants

Data of 44 participants in 22 pairs were analyzed. Within pairs, the participants were related to one another either as friends (14 pairs), couples (6), or siblings (2). All participants had pure tone hearing thresholds better than 20 dB HL. The average age was 22.2 years for female participants (n = 32) and 24.4 for the male participants (n = 12).

### Procedure

The experiment took place in a room of dimensions 4.11 × 2.59 × 2.54 m^3^ and reverberation time of T_30_ = 0.7 s. The pairs were fitted with headphones and boom microphones, as well as with their respective wireless receiver and transmitter along with two wireless motion trackers mounted to their headphones. The participants were instructed to have free conversations for two minutes after a cue was given, just before the playback of the noise stimulus began. Conversations were held either in a standing or a seated configuration. In the standing configuration, pairs began by standing in front of each other at a distance of 2.5 m and they were instructed to move freely, as needed, for comfortable conversation. In the seated configuration, the participants sat across a round table that was 0.76 cm in diameter and were instructed that they could position themselves where comfortable but they had to remain seated.

### Materials

Background noise stimuli were five real-world scenes from the Ambisonic Recordings of Typical Environments (ARTE) database^69^: Library (mean free-field level of 53 dB SPL), Living Room (63.3 dB SPL), Cafe (2) (71.7 dB SPL), and Train Station (77.1 dB SPL), Food Court (2) (79.6 dB SPL). Two additional noise stimuli were presented of a Party without background music (No Music Party; 85.0 dB SPL) and a Party with background music (Music Party; 92.0 dB SPL). A Bruel&Kjaer type 5128-C Head and Torso Simulator was used to transform these scenes into non-individualized binaural (in-ear) recordings that were then presented to participants (with no head tracking) over open headphones (Sennheiser HD-800), which allowed for nearly-transparent acoustic communication between participants^69,70^. The binaural recordings were low-pass filtered above 2000 Hz to match the slight acoustic attenuation of an external talker that was measured for this open headphone model^70^. The signals were generated in Matlab through a UFX RME sound card and a Sennheiser SR 300 IEM transmitter and were received by portable stereo wireless headphone receivers (Sennheiser EK 300 IEM) worn by the participants, which provided calibrated output level for the headphones (see^30^). Note that while the background noise environments used differ in other attributes beside their level, their long-term average level is their most dominant attribute, as was determined by a non-conversational rating task^52^. As such, we treat background noise level as continuous rather than parametric in the statistical modeling.

### Speech levels

Near-field speech signals were recorded using individual DPA d:fine™ FIO66 omnidirectional headset (boom) microphones that were placed near the participants’ mouths. Speech was recorded throughout the duration of each background noise (2 min) at a sampling rate of 44.1 kHz with 24 bits depth using the UFX RME sound card. The absolute output levels of the individual boom microphones were calibrated with reference to an omnidirectional microphone at a distance of 1 m, as described in^30,71^. To reduce the acoustic crosstalk between the two recording channels (i.e., talkers), the channels were separately analyzed in 30 ms Hann-windowed segments with 50% overlap and the quieter of the two speech segments was multiplied by zero before it was resynthesized using the overlap-add method^30^. The individual long-term speech levels were then derived using the method described in^72^, which excluded speech pauses or silent intervals. A final -1.87 dB correction was applied to provide sentence-equivalent levels^70^. This analysis was repeated for the 5 s of speech preceding and following a breakdown to derive the change in speech level of the other talker as an immediate response to the breakdown.

### Speech envelopes

To evaluate how the pairs adjusted their speech levels to the intrinsic temporal fluctuations of the background noise, their temporal envelopes were derived (in dB) by calculating the short-term levels within 2 s intervals. This temporal resolution seemed to provide the best compromise for capturing the relevant modulations in the speech and noise signals while avoiding excessive modulations due to individual speech utterances and pauses. However, we systematically varied this time window between 0.1 and 5 s but it had no significant effect on the main findings. For the noise signals, the envelope was derived separately for the left and right headphone signals via a Brüel & Kjær Type 4128 Head and Torso Simulator and then averaged (in the power domain) across ears to form a single envelope per environment. For the speech signals, the envelope was derived for each individual resynthesized talker signal (see above) and then the median value was taken across all subjects separately for each environment. As the speech level variations were driven by the noise level variations, a delay of approximately 2 s was observed and removed from the speech envelopes before any correlation analysis was performed.

### Motion capture

The head position of each participant was tracked using two motion trackers that were mounted on the outer left and right earpieces of the participant’s headphones without obstructing their grills. Tracked data of each marker were of six degrees of freedom—including both position (*x, y, z*) and rotation (*pitch, yaw, roll*)—which were obtained using a Polhemus Latus motion tracking system. Motion data from the markers were wirelessly detected by receptors that were mounted on 1.25 m stands with distance of 1.5 m between them that together provided full coverage of the participants’ positions at precision of ± 0.5 mm. The data from the markers were sampled at 120 Hz by the system which was synchronized to the audio playback and recording setup. The raw motion data were smoothed using a 10 Hz low-pass filter (fourth-order Butterworth) prior to the analysis (see^30^ for more details).

### Interpersonal distance

The interpersonal distance between participants was calculated for each trial with respect to the central, 3D-position of each participant’s head. At each time-step, we calculated the averaged (x, y, z) position of the motion tracking sensors attached to the left and right earpieces of the participants’ headphones. From the resulting ‘center-of-head’ (x, y, z) positional time series, a single interpersonal distance time series for each trial was calculated as the relative distance between the center-of-head position of participants in a pair. The overall average interpersonal distance time series for each condition (averaged over pair and trial) is displayed in Fig. 2A and B. The change in interpersonal distance (overall averages displayed in Fig. 2C for each trial) were calculated as the difference between the average interpersonal distance value observed during the first two seconds of a trial (i.e., starting interpersonal distance) and the averaged interpersonal distance value calculated during the last five seconds of a trial (i.e., final interpersonal distance). Finally, for the interpersonal distance breakdown analysis, the average interpersonal distance over the five seconds preceding and following a communication breakdown were employed for the analysis. A smaller interpersonal distance value following a communication breakdown compared to the interpersonal distance preceding the breakdown will indicate that the participants moved closer together immediately after a breakdown occurred.

### Coordination analyses

Due to the variable and stochastic nature of the participants’ body movements when conversing, Cross-Recurrence Quantification Analysis (CRQA) was employed to measure the magnitude and stability of the movement coordination that emerged between the pairs. As noted above, CRQA determines the dynamic similarity or covariance of two time-series trajectories independent of the distribution, stochasticity, or stationarity of the underlying data. In short, CRQA involves determining whether the states of two systems or behavioral trajectories are recurrent (close together, overlap) in reconstructed phase space and is known to be highly sensitive to the subtle space–time correlations that can occur between two motion trajectories (see^41,42,46^ and^7,45,46^ for tutorial review). As noted earlier, it has become one of the primary methods for assessing interpersonal movement coordination^9^ and, in particular, for the postural or movement coordination that emerges between individuals during conversation (e.g.,^7,43,44^).

To determine the similarity (%REC), structural organization (%DET), and stability (MAXLINE) of the movement coordination that occurred between participants we performed CRQA analysis on the participants’ center-of-head movement vector time series. We computed a single 3D movement displacement vector time series for each participant from their center-of-head time series and performed CRQA on the displacement vector^41^ time series for each trial. CRQA requires the selection of several key parameters for (i) defining the dimension of reconstructed phase space that movement trajectories are embedded in and (ii) determining what trajectory states in phase space are close enough together to be considered recurrent (see^73^ for details). For the data presented here, we employed an embedding dimension of six (determined using *false-nearest neighbors analyses*), a time-lag (T-lag) of 23 samples (determined using *average mutual information analysis*), and a recurrent point radius of 20% of the mean distance between points (see^41,45,46,73^ for more detail about the corresponding parameter analysis techniques). All data were also z-score normalized prior to analysis. Note that following recommended practice^41,45,46^, we validated that the results were not parameter dependent by conducting CRQA using embedding dimensions of 5 and 7, and T-Lags of 12, 18, 34, and 47, and radii of 15% and 25% of the mean distance between points, with the same pattern of results observed across all parameter settings.

### Communication breakdown analyses

All conversations (22 pairs × 7 noise environments × 2 talker configurations = 448 conversations) were professionally transcribed and made available in a single text file. The first author read the transcription and selected all interactions in which communication breakdowns occurred, coded as all instances of overt signaling of an other-initiated repair, which included open requests (e.g., ‘what’, ‘huh’), restricted requests (e.g., ‘who’) and restricted offers (e.g., ‘did you say manta rays’) and included all cases of repair-requests, regardless of whether they were standalone (i.e., independent occurrences), or stacked (e.g., multiple OIRs in one sequence). The respective time codes were documented to time align with the motion and speech acoustics.

While we anticipate that increasing background noise levels will increase communication breakdowns, here we are only capturing them through the overt verbal signaling of OIRs. This likely means there will be other cases of communication breakdowns that will not be reported. Indeed, it is well documented that listeners ‘let it pass’^74^ to not interrupt conversational flow and rely on context to recover the intended message, and also employ nonverbal communication such as head and body movement and facial gestures (e.g., ‘freeze look’^75^) to signal communication breakdown. It is also the case that our sample will contain instances of communication breakdowns that transpired not because of SNR problems related to the background noise level, but rather due to the mismanagement of turn-taking (still a potential speech intelligibility problem) and/or due to rapid topic shifts^63,76^. Importantly, we have also employed the capture all term ‘communication breakdown’ when discussing other-initiated repairs, and it is possible that there will be instances of repairs that were initiated not because of communication breakdown, but in fact, to prevent communication breakdown.

### Supplementary Information

Supplementary Table S1.

## Data Availability

Majority of the de-identified dataset for the reported results will be made available in the supporting online materials for replicating the main analyses reported in this paper. In particular, the transcripts and audio recordings of the conversations will not be made available as they contain identifiable, confidential detail. If readers are interested in additional analyses, please contact the corresponding author: kelly.miles@mq.edu.au.
