# Supplementary material for: Behavioral dynamics of conversation, (mis)communication and coordination in noisy environments
Source: Sci Rep. 2023 Nov 20;13:20271. doi: 10.1038/s41598-023-47396-y (PMC10662155; doi:10.1038/s41598-023-47396-y)
Supplement: Supplementary file 1 — Supplementary Table S1. [file 41598_2023_47396_MOESM1_ESM.docx]

**Supporting Information for**

**Behavioral Dynamics of Conversation, (mis)Communication and Coordination in Noisy Environments.**

Kelly Miles*****† ECHO Laboratory; Centre for Elite Performance, Expertise and Training, Department of Linguistics; Macquarie University Hearing, The Australian Hearing Hub, Macquarie University 0000-0002-4104-980X

Adam Weisser† ECHO Laboratory, Department of Linguistics; Macquarie University 0000-0003-2261-8349

Rachel W. Kallen, Centre for Elite Performance, Expertise and Training, School of Psychological Sciences, Macquarie University 0000-0002-0031-737X

Manuel Varlet, The MARCS Institute for Brain, Behaviour and Development, Western Sydney University 0000-0001-5772-2061

Michael J. Richardson, Centre for Elite Performance, Expertise and Training, School of Psychological Sciences, Macquarie University 0000-0001-9159-2774

Joerg M. Buchholz, ECHO Laboratory; Centre for Elite Performance, Expertise and Training, Department of Linguistics; Macquarie University Hearing, The Australian Hearing Hub, Macquarie University 0000-0001-6188-9761

† ﻿Shared first-authorship: both authors have contributed equally.

*Kelly Miles

**Email:**  [kelly.miles@mq.edu.au](mailto:kelly.miles@mq.edu.au)

**This PDF file includes:**

Table S1

Table S1. Statistical table of Pearson’s product-moment correlations for short term background noise level and speech level correlations.

|  |  |  |  |  |  |  |  |  |  |  |
| --- | --- | --- | --- | --- | --- | --- | --- | --- | --- | --- |
|  | **Background noise (dB SPL)** | **Talker  configuration** | **Pearson's r** | **Lower  95 CI** | **Upper  95 CI** | ***t* value** | ***p* value** | **Slope** |  |  |
|  | Library (53.0) | Sitting | 0.086 | -0.098 | 0.264 | 0.9218 | 0.36 | 0.05 |  |  |
|  |  | Standing | -0.117 | -0.293 | 0.067 | -1.2569 | 0.21 | -0.09 |  |  |
|  | Living Room (63.3) | Sitting | 0.281 | 0.104 | 0.441 | 3.1281 | **0.00** | 0.15 |  |  |
|  |  | Standing | 0.334 | 0.162 | 0.487 | 3.7844 | **0.00** | 0.18 |  |  |
|  | Cafe (71.7) | Sitting | 0.261 | 0.083 | 0.424 | 2.8922 | **0.00** | 0.25 |  |  |
|  |  | Standing | 0.073 | -0.111 | 0.252 | 0.4351 | 0.78 | 0.07 |  |  |
|  | Train Station (77.1) | Sitting | 0.752 | 0.661 | 0.822 | 12.194 | **0.00** | 0.37 |  |  |
|  |  | Standing | 0.73 | 0.632 | 0.805 | 11.419 | **0.00** | 0.36 |  |  |
|  | Food Court (79.6) | Sitting | 0.104 | -0.079 | 0.281 | 1.1214 | 0.26 | 0.11 |  |  |
|  |  | Standing | 0.315 | 0.141 | 0.47 | 3.542 | **0.00** | 0.30 |  |  |
|  | No Music Party (85.0) | Sitting | 0.566 | 0.428 | 0.679 | 7.3386 | **0.00** | 0.26 |  |  |
|  |  | Standing | 0.677 | 0.564 | 0.765 | 9.8087 | **0.00** | 0.33 |  |  |
|  | Music Party (92.0) | Sitting | 0.371 | 0.202 | 0.518 | 4.2632 | **0.00** | 0.38 |  |  |
|  |  | Standing | 0.513 | 0.364 | 0.636 | 6.3753 | **0.00** | 0.45 |  |  |
|  |  |  |  |  |  |  |  |  |  |  |
